# Activity of *Steinernema colombiense* in plant-based oils

**DOI:** 10.21307/jofnem-2020-072

**Published:** 2020-07-28

**Authors:** Gabriela Castruita-Esparza, Francisco Ángel Bueno-Pallero, Rubén Blanco-Pérez, Lídia Dionísio, Teodulfo Aquino-Bolaños, Raquel Campos-Herrera

**Affiliations:** 1 Instituto Politécnico Nacional, Centro Interdisciplinario de Investigación para el Desarrollo Integral Regional Unidad Oaxaca (CIIDIR-IPN-OAXACA), Hornos 1003 Colonia Nochebuena, Santa Cruz Xoxocotlán, Oaxaca, CP 71230, México; 2 UDIT MED – Mediterranean Institute for Agriculture, Environment and Development, Pólo, Universidade do Algarve, Campus de Gambelas, Ed 8, 8005-139, Faro, Portugal; 3 Instituto de Ciencias de la Vid y del Vino (ICVV), Gobierno de La Rioja, CSIC, Universidad de La Rioja, Ctra Burgos Km 6 Salida 13 Lo-20, 26007, Logroño, Spain

**Keywords:** *Beauveria bassiana*, Biological control, Coconut oil, Olive oil, *Steinernema*, Temperature

## Abstract

Entomopathogenic nematodes (EPNs) are excellent biological control agents. Although traditionally EPN application targeted belowground insects, their aboveground use can be supported if combined with adjuvants. We hypothesized that EPN infective juveniles (IJs) could be combined with plant-based oils as adjuvants, without decreasing their efficacy against insect larvae under various scenarios. Specifically, our objectives were to evaluate the activity of *Steinernema colombiense* (Nematoda: Steinernematidae) when mixed with two plant-based oils (coconut and olive oils) and maintained at different temperatures and times, or combined with entomopathogenic fungi. First, we evaluated how these oils affected IJ survival and virulence against last instar *Galleria mellonella* (Lepidoptera: Pyralidae) larvae when maintained at five different temperatures (4, 8, 14, 20, and 24°C) and five incubation times (1, 3, 7, 14, and 21 days), using water as control treatment. Second, we evaluated virulence when combined with these two oils as well as with water (control) and combined with the entomopathogenic fungi (EPF), *Beauveria bassiana* (Hypocreales: Clavicipitaceae). Infective juvenile survival was higher in coconut than olive oil and water mixtures up to 7 days at 4°C. Conversely, olive oil supported higher larval mortality than coconut oil at 4 to 20°C and 14 days. Similarly, the number of days needed to kill insect larvae increased at extreme temperatures (4 and 24°C) after 14 days. Finally, the EPN + EPF combination showed an additive effect compared to EPN and EPF single treatments. Our findings indicate that our plant-based oil mixtures maintain viable IJs at moderate temperatures and up to 7 to 14 days, and can be used in single EPN mixtures or combined with EPF.

Entomopathogenic nematodes (EPNs) in the genera *Steinernema* and *Heterorhabditis* are well-known biological control agents used against many arthropod species (Campos-Herrera, 2015; Lacey et al., 2015). They selectively search for insect hosts and kill them within 2 to 3 days with the aid of mutualistic bacteria of the genera *Xenorhabdus* and *Photorhabdus*, respectively (Adams et al., 2006; Dillman et al., 2012). Their worldwide distribution in soils (Kaya et al., 2006) and the availability of commercial products (Lacey et al., 2015) make the EPNs excellent agents to employ them in integrated pest management (IPM) programs and under organic production (Campos-Herrera, 2015).

Formulation and application technique are key aspects of the use of EPNs as biological control agents. The selection of the best formulation and application technique may differ depending on the target pest and its location, above or belowground (Hiltpold, 2015). The most widespread practice for EPNs use in biocontrol agent is the massive application of infective juveniles (IJs) stages in belowground agroecosystems, where they are naturally adapted (Lacey et al., 2015). Numerous substrates such as vermiculite, clay, activated charcoal, polyacrylamide, alginate capsules, or simple water-dispersible granules have been tested as formulation agents with variable shelf life and storage limitations (Hiltpold, 2015; Ramakuwela et al., 2015; Kary et al., 2018; Leite et al., 2018; Touray et al., 2020). Then, EPN is applied in the soil by creating a simple suspension in water, which is easy to manage and cheap for growers to use. Survival and activity of EPNs on soils will differ depending on the substrate selected for their formulation and the environmental conditions of storage (temperature, humidity, and time) (Grewal, 2000; Kim et al., 2015; Ramakuwela et al., 2015; Leite et al., 2018; Touray et al., 2020).

EPN application for targeting aerial pests requires extra adjustments to keep them located on the target plant part (Brusselman et al., 2012; Shapiro-Ilan and Dolinski, 2015). Often aerial application approaches include adjuvants or surfactants to enhance IJ survival under stressing factors, such as desiccation and UV exposure (Schroer and Ehlers, 2005; Beck et al., 2013; De Waal et al., 2013; Hiltpold, 2015; Dito et al., 2016; Noosidum et al., 2016), but also to maintain virulence and reduce drippage from leaves. Polymers as Zeba and sprayable fire-gels are among the synthetic compounds tested for this purpose (Lacey et al., 2010; Shapiro-Ilan et al., 2010, 2015; De Waal et al., 2013; Portman et al., 2016; Noosidum et al., 2016). Also, mixtures based on plant-based oils reported promising results (Krishnayya and Grewal, 2002; Qiu et al., 2008; Moreira et al., 2013; Monteiro et al., 2014; Alves et al., 2017; Aquino-Bolaños, Morales-García and Martínez-Gitiérrez, 2019; Aquino-Bolaños, Ruiz-Vega, Ortiz Hernández and Jiménez Castañeda, 2019), expanding the range of specific physical-chemical properties to consider. For example, since certain plant-based oils such as coconut oil remain solid at relative high temperatures (~24°C), this could be of interest for producing stable shipments. Hence, exploring the potential of using oil-based adjuvants such as olive and coconut oils, both often accessible in regular stores, would provide an alternative for local EPN producers.

There are several options to enhance the efficiency of EPN formulations and application approaches. An example could be the additions of chemical compounds such as pheromones (Shapiro-Ilan et al., 2019). Also, the simultaneous application of EPNs with other beneficial soil organisms has shown enhanced biocontrol and plant protection activity. Previous studies have reported successful co-applications with entomopathogenic fungi (EPF), arbuscular mycorrhizal soil fungi, and bacteria in the genus *Pseudomonas* (Shapiro-Ilan et al., 2004; Molina-Acevedo et al., 2007; Ansari et al., 2008; Imperiali et al., 2017; Jaffuel et al., 2019). This co-application approach is a promising tool for sustainable agriculture that, once optimized, could save costs and time to growers. The EPF *Beauveria bassiana* (Hypocreales: Clavicipitaceae), which occurs in natural and agricultural soils worldwide, is one of the most prominent biological control agents used among commercial products (Lacey et al., 2015), and thus, can be an excellent candidate for combinations of this kind.

Regional programs IPM often promote local EPN productions (San-Blas et al., 2019). In this context, new systems that guarantee high IJ survival and virulence after medium-term storage must be developed without significantly increasing costs and with relative accessible products. Herein, we explored two plant-based oils used as model of possible adjuvant: coconut (*Cocos nucifera*) and olive (*Olea europaea*). We selected the EPN species *Steinernema colombiense*, described by López-Núñez et al. (2008). This nematode is naturally occurring in several countries of Latin America, and has shown promising results against several below and aboveground insect pests (Delgado-Ochica and Sáenz-Aponte, 2012; Rosero-Guerrero et al., 2012; Aristizábal et al., 2015). We hypothesized that coconut and olive oils, as models plant oil-based adjuvants, can be combined with EPN to be used as mixing media, single applied or combined with the EPF species *B. bassiana*, without any deleterious effect or negative impact on virulence. Therefore, the objectives of this study were: (i) to evaluate the survival and virulence of *S. colombiense* against last instar larvae of *Galleria mellonella* (Lepidoptera: Pyralidae) when combined with coconut and olive oils as model adjuvants at different temperatures and times, and (ii) to evaluate the efficacy of using these plant-based oil combined with *S. colombiense* and *B. bassiana*.

## Material and methods

### Organisms, oils, and substrates

We conducted the experiments by employing *S. colombiense* isolated from Mexico and donated by Centro Interdisciplinario de Investigación para el Desarrollo Integral Regional, Unidad Sinaloa – CIIDIR Sinaloa (México) (ITS region, GenBank accession number MG551678). The EPF *B. bassiana* was isolated in Algarve (Portugal) (GenBank access number MG515530, Bueno-Pallero et al., 2018). The insect *Galleria mellonella* (Lepidoptera: Pyralidae) was used as insect hosts, reared at the University of Algarve (Portugal). The nematodes were regularly refreshed in vivo using last instar *G. mellonella* as the host (Woodring and Kaya, 1988). The IJs were recovered upon emergence and stored in mineral water at 14°C. These were used within 2 weeks of harvest (Bueno-Pallero et al., 2018). *Beauveria bassiana* was isolated from a naturally infected adult of *Rhynchophorus ferrugineous* (Coleoptera: Curculionidae), cultured on 90-mm diam. petri dishes with potato dextrose agar (PDA, Biokar) at 25 ± 1°C, avoiding sub-culture more than once during the study (Shapiro-Ilan et al., 2004), and stored at 4°C until use (Goettel and Inglis, 1997). The coconut and olive oils tested were obtained from commercial products (San Lucas^®^ and Carbonell^®^, respectively), and maintained in cold at dark conditions until use. Finally, we used pure mineral sand (Vale do Lobo, Loulé, Portugal) as a substrate in the infectivity test. To prepare the sand for each experiment, it was washed several times with running water, autoclaved for 1 h (two times in two consecutive days as suggested by Elhady et al., 2018), oven-dried at 40°C with ventilation, and stored in laboratory conditions at least for a week before being used (Chiriboga et al., 2017).

### Combination of entomopathogenic nematodes with plant-based oils at different temperatures and times

We assessed for the survival and virulence of *S. colombiense* combined with coconut oil, olive oil, or water (control) at five temperatures (4, 8, 14, 20, and 24°C) and five incubation times (1, 3, 7, 14, and 21 days). We used 24-wells plates (Falcon Multiwell, 24-well Polystyrene, Corning Incorporated-Life Sciences, Duham, USA), designating eight wells per treatment (coconut, olive, or water) per plate. The treatments consisted of 20 IJs released in 200 μl final volume per well of coconut/olive oil mix (60 μl of oil and 140 μl of water) or distilled water as a negative control (Glazer and Lewis, 2000). Because the oils do not mix with water, we prepared the corresponding mixtures by stirring the proportional quantities of water/nematodes and oils in a big beaker with a stirring bar. The oil mixtures were applied when the combination appeared well mixed and the size of the oil particles were minimal. In addition, in the case of the coconut oil, we warmed this suspension to 25 to 28°C to ensure that the oil was liquid since the coconut is solid at temperatures below 24°C (https://en.wikipedia.org/wiki/Coconut_oil). The plates were closed with parafilm and stored in the corresponding temperature in a plastic container with moistened towels to prevent evaporation. We estimated nematode survival rates by counting the number of live IJs per well at the inoculation time and after incubation times, touching straight nematodes to verify their capacity to move. In the case of the coconut treatments, we warmed for few minutes in a metal plate at 25°C to merge the oil. After the nematode survival estimation, we added 2 g of sterilized sand and one last instar *G. mellonella* larva per well to evaluate the EPN infectivity, incorporating additional plates with adjuvant treatments but no nematodes as controls. We revised daily larval mortality for an incubation period of five days at 24°C in dark conditions (Dillman et al., 2012). To verify that mortality was due to EPN activity, we transferred all insect cadavers individually to White traps (White, 1927) and incubated them in the same conditions, until confirming nematode emergences. The whole experiment was performed twice using new nematodes, insects, and adjuvants.

### Combination of entomopathogenic nematodes and fungi with plant-based oils

To determinate the nature of the interaction between *S. colombiense* and *B. bassiana*, we first evaluated single applications at different concentrations (Koppenhöfer and Grewal, 2005). We employed 24-wells plates, inoculating 100 μl of each suspension per well (*n* = 20 per treatment, two independent trials). The EPN concentrations tested were 0, 1, 5, 10, 20, 50, and 100 IJs/well, all applied from volumetric suspension accordingly adjusted, except 1 and 5 IJs/well, handpicked (Blanco-Pérez, et al., 2019). For *B. bassiana*, we prepared conidia suspensions using a sterile swab to transfer the conidia from PDA massive production plates to a Falcon^®^ tube with sterile half-strength Ringer solution and 0.05% Tween 80 (Klingen et al., 2002). The concentrations used (0, 10^0^-10^8^ conidia) were estimated by counting in a hemocytometer (Neubauer improved) after the conidia suspension was homogenized (Vortex^®^) for 2 to 3 min (Bueno-Pallero et al., 2018). After EPN or EPF application of the corresponding concentration, we placed 2 g sterilized soil and one last instar *G. mellonella* larvae per well. All plates were closed with parafilm, incubated at 24°C in dark conditions in a plastic container with moistened towels to prevent evaporation. Larval mortality was checked daily for a week. In addition, as technical control for EPF, we inoculated PDA plates with all the prepared conidia concentrations for ensuring viability.

We estimated that the inoculation of 2 IJs of *S. colombiense* and 1 × 10^7^ spores of *B. bassiana* per well was required to kill ~50% of the *G. mellonella* larvae. We added those quantities in a final volume of 200 μl per well of the coconut/olive oil mixture (60 μl of oil and 140 μl of water) or distilled water (control). As described before, the oil mixtures were prepared in a big beaker with a stirring bar and applied when the combination appeared well mixed. In addition, in the case of the coconut oil, we warmed this suspension to 25 to 28°C. The corresponding suspensions were inoculated in 24-wells plates as previously described (eight wells per adjuvant, two 24-plates per organism: EPN, EPF, EPN + EPF, and none organism as control). Then, we added 2 g of sterilized sand and one last instar *G. mellonella* per well. Plates were closed with parafilm, incubated at 24°C in the dark, and introduced in a plastic container with moistened towels to prevent evaporation. Larval mortality was monitored daily for 5 days. As before, we transferred all insect cadavers individually to modified White traps and incubated them in the same conditions, until confirming nematode/fungi emergences. The whole experiment was performed twice using new nematodes, fungi, insects, and adjuvants.

### Statistical analyses

First, the appropriate concentration for the EPN and EPF co-application test was estimated by Probit analysis (SPSS 25.0), pooling the data of two independent trials (data not shown). We calculated the percentage of live (mobile) IJs after the incubation times for each treatment, considering the total mobile IJs before and after application. Similarly, we estimated the virulence for each treatment by calculating the frequency of larval mortality and the number of days needed to kill. Before statistical analysis, percentage values were arcsine transformed, and data from different trials were combined after checking the statistical similarity of the results (data not shown). We ran generalized linear models (GLM, *P* < 0.05) for the analysis of the variables percentage of alive IJs, larval mortality, and days needed to die. For the first experiment, the factors tested were the adjuvant (three levels: control/water, coconut oil, and olive oil), the temperature (five levels: 4, 8, 14, 20, and 24°C), the time (five levels: 1, 3, 7, 14, and 21 days), and their interactions. In addition, we performed an individual one-way ANOVA and Tukey test (*P* < 0.05) for each temperature and time of incubation to disentangle the specific impact of the adjuvants. For the second experiment, EPN–EPF combination, the factors tested were the adjuvant (three levels: control/water, coconut oil, and olive oil), organisms applied (four levels: EPNs, EPF and EPNs + EPF, and control) and their interactions.

We followed the formulae proposed by Shapiro-Ilan et al. (2004) and Ansari et al. (2010) to evaluate the nature of the EPN–EPF interaction (antagonistic, no-interaction/additive, or synergistic), comparing the expected and observed mortalities for each adjuvant (coconut/olive oil or distilled water). The expected mortalities (*M*_*E*_) were calculated as *M*_*E*_ = *M*_*T*1_ + [*M*_*T*2_ × (1 − *M*_*T*1_)] when EPNs and EPF were combined. We run a *χ*^2^ test for the expected and observed mortalities [i.e. *χ*^2^ = (*M*_*T*1*T*2_ − *M*_*E*_)^2^/*M*_*E*_, where *M*_*T*1*T*2_ is the observed mortality for each organism single applied]. Those values were matched with the *χ*^2^ table for 1 degree of freedom (*P* = 0.05) so that *χ*^2^ < 3.8415 indicated additive interaction and *χ*^2^ > 3.8415 non-additive (antagonist or synergist) interaction. Thus, the interaction was considered synergistic if *M*_*T*1*T*2_ − *M*_*E*_ > 0, and antagonistic if *M*_*T*1*T*2_ − *M*_*E*_ < 0 (Shapiro-Ilan et al., 2004; Ansari et al., 2010).

We performed all the analyses with SPSS 25.0 (SPSS Statistics, SPSS Inc., Chicago, IL, USA). We used least-square means ± S.E. as descriptive statistics.

## Results

### Impact of temperature and time on the survival and virulence of the entomopathogenic nematode combined with plant-based oils

**Figure 1:**
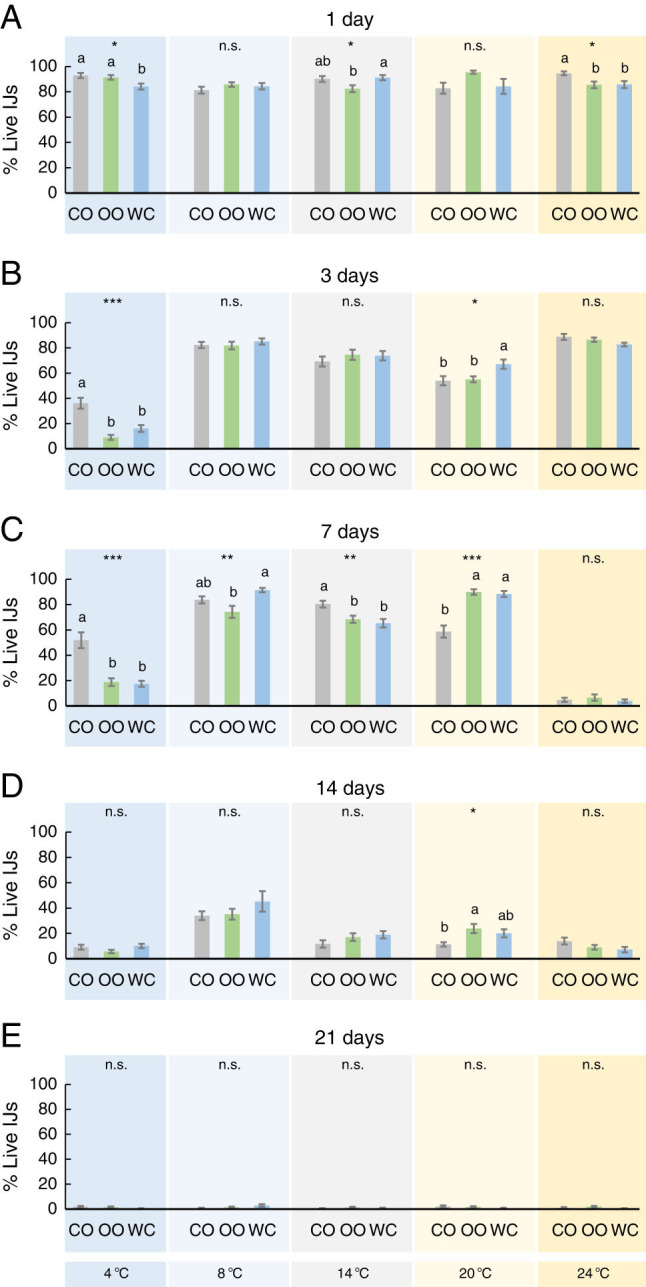
Effect of three adjuvants employed in *Steinernema colombiense* combination, coconut oil (CO), olive oil (OO), and distilled water (WC), on the percentage of live (mobile) infective juveniles (IJs) previously incubated at five different temperatures (4-24°C) and for five different times (1-21 days). Asterisks indicate significant differences within treatment comparisons at *P < 0.05; **P < 0.01; ***P < 0.001, and n.s., not significant. Different letters indicate significant differences in Tukey’s test (HSD). Values are least-square means ± SE.

EPN survival measured as percentages of live IJs resulted in statistically significant for all factors (adjuvant and temperature and time of incubation) and their interactions except for adjuvants (Table 1, Fig. 1). We analyzed all the three factors individually to disentangle for specific effects (Fig. 1). Overall, we observed most of the statistical differences among adjuvants for short periods (1-7 days). Particularly, we found more live IJs in coconut mix than in water after 1 day and at extreme temperatures (4 and 25°C) (Fig. 1A), a trend also observed at 4°C after 3 and 7 days (Fig. 1B, C) and at 14°C for day 7 (Fig. 1C). Conversely, for moderate temperatures (8, 14, and 20°C), higher percentages of live IJs were more often reported for water than for plant-based oils (Fig. 1A-C). The temperature of 25°C drastically reduced IJ survival after 7 days, independently of the adjuvant used (Fig. 1C-E). In any case, long periods considerably decreased the percentage of live IJs (below 20 and 2% for 14 and 21 days, respectively; Fig. 1D, E), only significantly higher for olive oil than for coconut oil for 14 days at 20°C (Fig. 1D).

**Table 1.** Statistical analysis of the effect of three factors (adjuvants, temperature and time of storage) and their interactions (GLM, *P* < 0.05) in the infective juvenile (IJ) survival measured as percentage of live IJ, and its virulence measured as larval mortality and number of days to kill the insects.

Fixed factors	% Live IJs	% Dead larvae	No. days to die
Adjuvant (Adj)	*F*_2, 1,125_ = 1.53; *P* = 0.217	*F*_2, 1,125_ = 15.31; ***P*** < **0.001**	*F*_2,786_ = 0.69; *P* = 0.513
Incubation temperature (Temp)	*F*_4, 1,125_ = 228.95; ***P*** < **0.001**	*F*_4, 1,125_ = 49.31; ***P*** < **0.001**	*F*_4,786_ = 9.24; ***P*** < **0.001**
Incubation time (Time)	*F*_4, 1,125_ = 2,309.61; ***P*** < ** 0.001**	*F*_4, 1,125_ = 274.73; ***P*** < **0.001**	*F*_4,786_ = 185.90; ***P*** < **0.001**
Adj × Temp	*F*_8, 1,125_ = 17.40; ***P*** < **0.001**	*F*_8, 1,125_ = 2.67; ***P*** = **0.007**	*F*_8, 786_ = 1.86; *P* = 0.063
Adj × Time	*F*_8, 1,125_ = 2.31; ***P*** = **0.018**	*F*_8, 1,125_ = 7.39; ***P*** < **0.001**	*F*_8, 786_ = 3.26; ***P*** = **0.001**
Tem × Time	*F*_16, 1,125_ = 136.59; ***P*** < **0.001**	*F*_16, 1,125_ = 19.43; ***P*** < **0.001**	*F*_16, 786_ = 10.89; ***P*** < **0.001**
Adj × Temp × Time	*F*_32, 1,125_ = 5.52; ***P*** < **0.001**	*F*_32, 1,125_ = 1.42; *P* = 0.061	*F*_32, 786_ = 2.11; ***P*** = **0.001**

**Figure 2:**
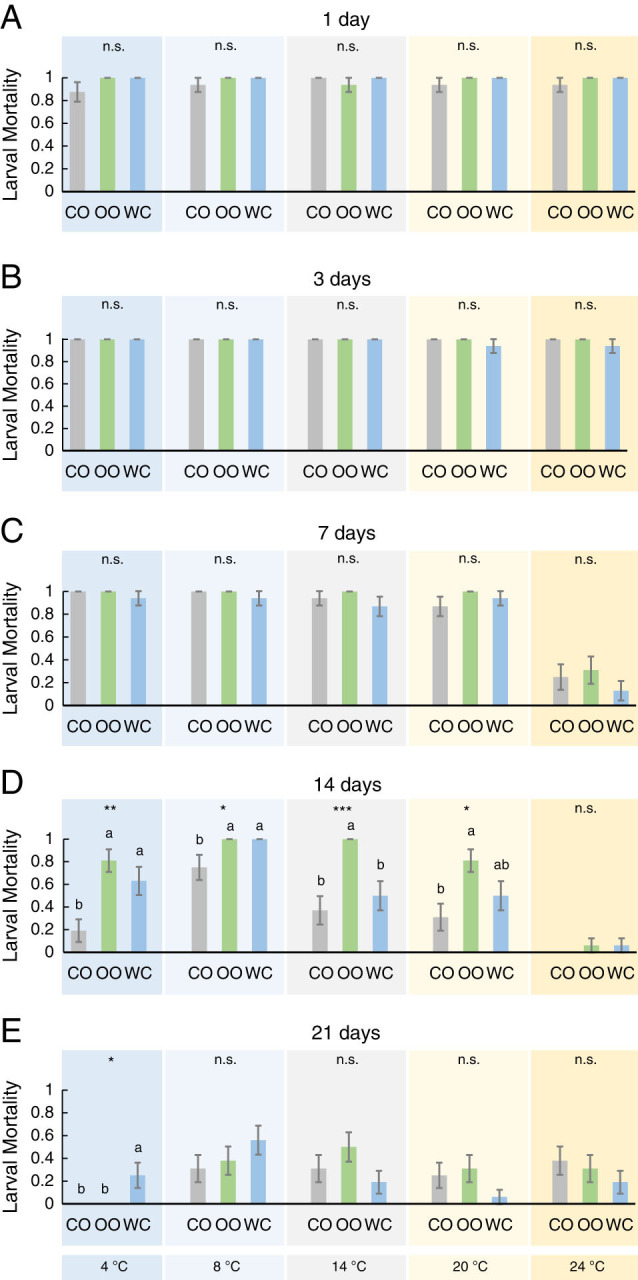
Effect of three adjuvants employed in *Steinernema colombiense* combinations, coconut oil (CO), olive oil (OO), and distilled water (WC), on the frequency of larval mortality of last instar *Galleria mellonella* previously incubated at five different temperatures (4-24°C) and for five different times (1-21 days). Asterisks indicate significant differences within treatment comparisons at *P < 0.05; **P < 0.01; ***P < 0.001, and n.s., not significant. Different letters indicate significant differences in Tukey’s test (HSD). Values are least-square means ± SE.

Larval mortality was statistically significant for all factors and their interactions except for the adjuvant×time×temperature triple interaction (Table 1, Fig. 2). We observed in the individualized analysis that larval mortality was over 85% for 1 to 7 days (except for 7 days at 25°C) without differences among adjuvants (Fig. 2A-C). For 14 days, we recorded significantly lower larval mortalities for coconut oil than for olive oil (at 4-20°C) and for water (at 4 and 8°C) (Fig. 2D). At 24°C, 7 or more days of incubation resulted in a considerable reduction of the larval mortality (~50% or less; Fig. 2C-E). After 21 days, larval mortality was significantly higher for water than for plant-based oils at 4°C (Fig. 2E).

**Figure 3:**
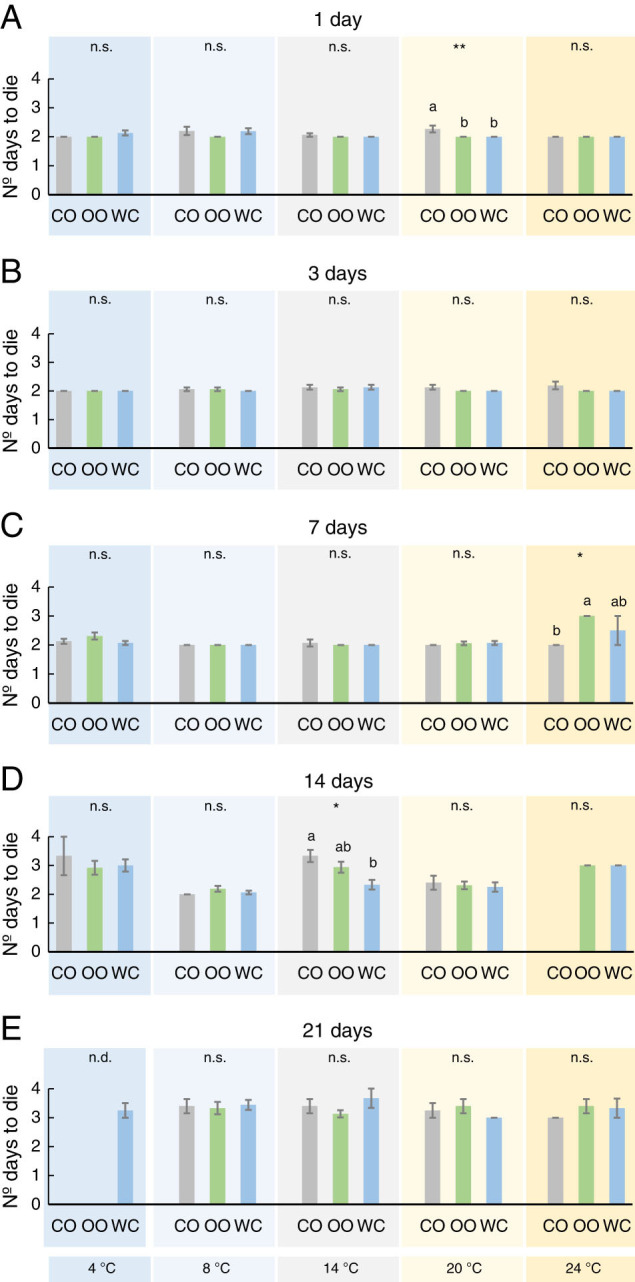
Effect of three adjuvants employed in *Steinernema colombiense* combinations, coconut oil (CO), olive oil (OO), and distilled water (WC), on the number of days needed to kill last instar *Galleria mellonella* larvae previously incubated at five different temperatures (4-24°C) and for five different times (1-21 days). Asterisks indicate significant differences within treatment comparisons at *P < 0.05; **P < 0.01; ***P < 0.001, and n.s., not significant. Different letters indicate significant differences in Tukey’s test (HSD). Values are least-square means ± SE.

The number of days needed to kill the *G. mellonella* larvae was statistically significant for all factors and their interactions except for the adjuvant employed and its interaction with temperature (Table 1). We found few differences among adjuvants in the individualized analyses. Essentially, IJs killed slower in the coconut mix for 1 and 14 days at 20 and 14°C, respectively (Fig. 3A, D), but faster for 7 days at 25°C (Fig. 3C). Overall, IJs stored up to 14 days needed 2 days on average to kill (Fig. 3A-D), while ~3 days were needed for IJs stored for 21 days (Fig. 3E) or for 14 days at extreme temperatures (4 and 24°C; Fig. 3D).

### Combination of entomopathogenic nematodes and fungi with plant-based oils

**Figure 4:**
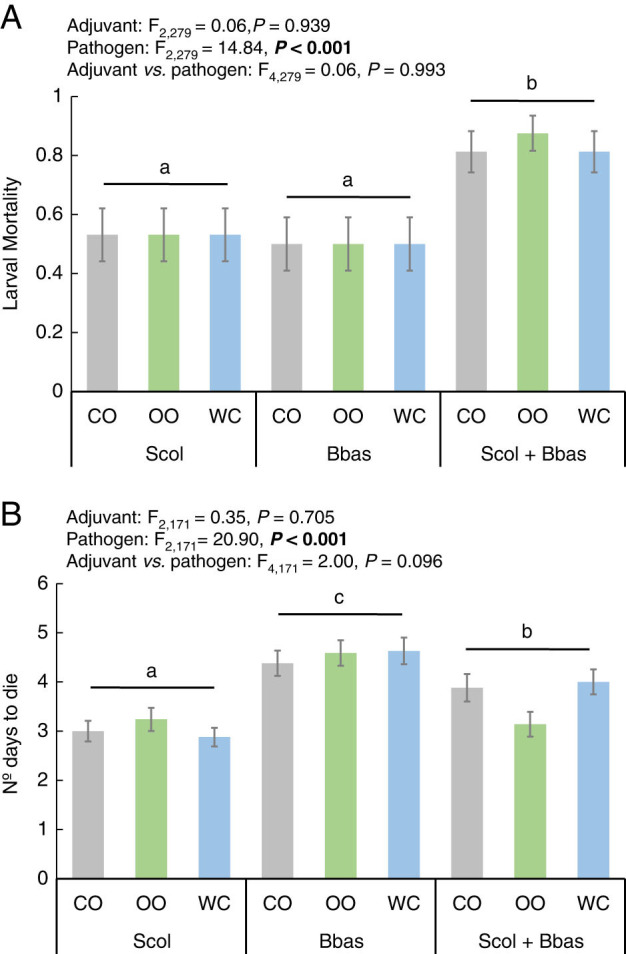
Virulence of *Steinernema colombiense* (Scol) and *Beauveria bassiana* (Bbas), single applied or combined (Scol+Bbas), combined with coconut oil (CO), olive oil (OO), and distilled water (WC). A: Frequency of last instar *Galleria mellonella* larval mortality. B: Number of days needed to kill the insect larva. Different letters indicate significant differences in Tukey’s test (HSD). Values are least-square means ± SE. Significant differences for GLM analysis (*P* < 0.05) are highlighted in bold.

The entomopathogenic activity was not affected by the adjuvant employed, but for the organisms (EPN, EPF, or EPN + EPF) applied (Fig. 4). The combined application of both entomopathogenic organisms, *S. colombiense* and *B. bassiana*, resulted in significantly higher larval mortalities than single application (Fig. 4A). The evaluation of the EPN–EPF interaction confirmed an additive effect for their co-application independently of the adjuvant employed (Table 2). Additionally, the combined application of both entomopathogens significantly reduced the number of days needed to kill insect larvae than *B. bassiana* single applied (Fig. 4B). However, those numbers remained significantly higher than recorded for EPNs single applied except if formulated with olive oil (Fig. 4B).

**Table 2.** Interactions of the co-application of entomopathogenic nematodes and entomopathogenic fungi formulated in different adjuvants for the suppression of *Galleria mellonella* larvae after.

Adjuvant	Observed mortality^a^	Expected mortality^b^	*χ*^2^	Interaction
Distilled water	81.3	76.6	0.28	Additive
Cocoa oil	81.5	76.6	0.31	Additive
Olive oil	87.8	76.6	1.58	Additive

**Notes:** ^a^Observed mortality (%), average of 16 replicates in two trials (32 total); ^b^expected mortality (%), calculated *M*_*E*_ = *M*_*T*1_ + *M*_*T*2_ × (1 − *M*_*T*1_) for combination of two organisms applied on the larvae, where *M*_*T*1_ and *M*_*T*2_ are the mortalities from *Steinernema colombiense* and *Beauveria bassiana* single applied, respectively.

## Discussion

As we hypothesized, overall, *S. colombiense* did not suffer significant deleterious effects in survival nor in infectivity when combined with the two plant-based oils than in treatment with water. Specifically, we recorded similar survival *S. colombiense* of IJs formulated with coconut and olive oils and water (control treatment) for 3 to 7 days at 8 to 20°C. In agreement with Kary et al. (2018), the adjuvant employed and time and temperature resulted in factors that significantly interact with nematode survival rates. Kary et al. (2018) reported that one month at 24 to 25°C reduced the survival rates of *H. bacteriophora* IJs (below 50%) for some adjuvants compared to lower temperatures (over 70% at 8-15°C). We also recorded over 50% survival rates for *S. colombiense* IJs stored at mid-temperatures (8, 14, and 20°C), but only up to 7 days. IJ survival reduced significantly after 14 days, even below 2% at 21 days. EPN species-specific differences could explain the disparities observed between these studies (Shapiro-Ilan and Dolinski, 2015). Also, studies that investigate plant-based oil mixtures registered contrasting results. For example, over 95% of *S. feltiae* IJs survived if mixed with plant-based oil (Oxiquímica Agrociência, Ltda) and stored at 24°C for 5 days (Moreira et al., 2013). Under similar conditions (7 days at 24°C), *S. colombiense* IJs survived no more 5% independently of the adjuvant tested. This difference can be due to the fact that Moreira et al. (2013) employed tanks with specific pressure and a mix of 1% of the adjuvants, while our proportion was significantly higher and no pressure was applied. In addition, we did not employ any emulsifier, which could contribute to the formation of two layers, one below with water and the nematodes and one above with the oils. These two layers could limit the air exchange in the well, and hence, could have promoted the reduction of the IJs survival after 7 days. On the other hand, the survival of *S. colombiense* after 7 days at 20°C were higher than recorded for *S. websteri* mixed with citronella (*Cymbopogon citratus*) and red cedar (*Juniperus virginiana*) at similar exposure conditions (6 days at 22°C), but lower if compared with the results obtained for *S., carpocapsae* in the same study (Aquino-Bolaños, Morales-García and Martínez-Gitiérrez, 2019). Finally, Alves et al. (2017) showed that certain plant-based oils could be incompatible with EPN formulations. In their study, the combination of *Heterorhabditis* sp. CB40 with TEK-F^®^ resulted in 16.4% IJ survival, while the results with Aureo^®^ were similar that water controls (97.4 and 93.2%, respectively). Consequently, different plant-based oils can have positive, negative, or neutral effects (as for the coconut and olive oil mix showed in our study). Hence, testing the efficacy of the particular combination of EPN species with a new plant-based oil adjuvant is highly recommended before application.

Similarly, nematode virulence (larval mortality and time to kill insect larvae) considerably decreased from 14 days. From 1 to 7 days, independently of the adjuvant employed, we recorded over 80% of larval mortalities at most of the temperatures. This high larval mortality can be explained by the fact that only one nematode is required to survive to kill an insect (Stock, 2015). Aquino-Bolaños, Morales-García and Martínez-Gitiérrez (2019) reported similar values for the EPN species *S. wesbteri* and *H. bacteriophora* formulated with *C. citratus* and *J. virginiana*, while *S. carpocapsae* formulated with *C. citratus* only provided 60% of larval mortality. Conversely, we obtained less than 50% of larval mortality for IJs stored for 7 days at 24°C, which is a result that contrasts with the 100% mortality reported by Moreira et al. (2013) for plant-based oil adjuvants under similar conditions (5 days at 24°C). The larval mortalities of *Hedypathes betulinus* (Coleoptera: Cerambycidae) registered by Alves et al. (2017) were ◻80% and below 20% for *Heterorhabditis* sp. formulated with Aureo^®^ and TEK-F^®^, respectively. Finally, IJs mixed with coconut oil improved their virulence (survival and time to kill rates) from 1 to 7 days at low and high temperatures (4 and 24°C), although without implying higher larval mortality rates. In the case of olive oil mixtures, we recorded higher mortality rates at 14 days at 14°C.

We demonstrate the compatibility of both plant-based oils for the combination with the EPN species *S. colombiense* and the EPF *B. bassiana*. As shown for other EPN–EPF combinations without adjuvant combination (Wu et al., 2014), the combination of both entomopathogens resulted in an additive effect, independently of the adjuvant tested. Previous studies have shown that the nature of their interaction (additive, synergic, or antagonistic) is species specific, but also the concentration and the timing of the applications affect it. For example, Ansari et al. (2008) reported that the combined applying of *Metarhizium anisopliae* with different EPN species resulted in synergistic effects. In the case of *H. bacteriophora,* this synergic effect was observed independently of the timing of the application, whereas for *S. kraussei* and *S. feltiae* it was synergistic only when applied simultaneously or after 1 to 2 weeks of fungal inoculation, respectively. Wu et al. (2014) reported additive interactions when *H. bacteriophora* and *H. megidis* were simultaneously applied with *B. bassiana* and *M. anisopliae.* Finally, other studies found that, except for a few exceptions, many EPN–EPF interactions resulted in antagonistic interactions (Shapiro-Ilan et al., 2004; Acevedo et al., 2007).

We conclude that this study supports the use of coconut and olive oils as adjuvants, with certain shelf-life properties depending on the time and temperature. This can be of interest for local EPN producers that might need to store and ship their IJs under certain conditions. Moreover, we consider the additive effect of the simultaneous combination of EPN–EPF, and the lack of impact of the plant-based oil tested in their performance to be very promising. This approach could reduce the number of applications in the field and, consequently, the cost for growers. Further studies are required to confirm its feasibility. Additionally, the fact that the combination of EPN–EPF with olive oil resulted in larval mortality of 88% instead of ~50% recorded for the single EPN applications, but at similar times (~3 days), could be of interest when field treatments may require high mortalities at a short time. However, further studies are needed to deeper investigate the practical use of EPNs, single applied or combined with EPF, mixed with oils used herein or other plant-based oils, and at higher concentrations for biocontrol targeting aerial pests.
